# Is testosterone involved in low female sexual desire?

**DOI:** 10.20945/2359-3997000000153

**Published:** 2019-07-12

**Authors:** Carmita H. N. Abdo

**Affiliations:** 1 Universidade de São Paulo Departamento de Psiquiatria Faculdade de Medicina Universidade de São Paulo Brasil Psiquiatra, Professora Livre- -Docente do Departamento de Psiquiatria da Faculdade de Medicina da Universidade de São Paulo (FMUSP). Fundadora e Coordenadora do Programa de Estudos em Sexualidade (ProSex) do Instituto de Psiquiatria do Hospital das Clínicas da FMUSP. Presidente da Associação Brasileira de Psiquiatria (ABP), São Paulo, SP, Brasil; Universidade de São Paulo Instituto de Psiquiatria do Hospital das Clínicas FM USP Brasil; Associação Brasileira de Psiquiatria São Paulo SP Brasil

We are living an era of growing longevity due to the new technological and medical advances. However, we are also going through a time of unsuitable habits, in which women are increasingly exposed to stress, little physical activity, few hours of sleep, unbalanced diet, result of these same resources and/or the imperative need to access them.

Since the beginning of the 1900s, the higher life expectancy gave rise to a new population contingent: postmenopausal women (1). It should be remembered that menopause, besides being universal (occurs with all women in their 50s) is physiological and despite the increasing amount of women who do not produce estrogen anymore, the ‘’replacement” of this hormone is not a consensus among experts.

In parallel with this lack of consensus, the use of testosterone to improve low female sexual desire has been another arena, including women of all ages.

A recently published systematic review on benefits and harms of testosterone administrated to male sexual dysfunction concluded that testosterone therapy can be considered for men with low or low-normal testosterone levels and issues with sexual desire, erectile function and satisfaction derived from overall sexual life. The exact formulation, dosage and duration of treatment still needs to be clarified, the safety profile also remains unclear (2).

A higher incidence of low sexual desire and low sexual satisfaction, as well as dyspareunia, characterizes the sex life of a significantly larger proportion of women, compared with those in the premenopausal period. Hypoactive sexual desire dysfunction (HSSD) includes some symptoms with a duration of at least 6 months and is associated with clinically significant personal distress (frustration, grief, guilt, incompetence, loss, sadness, sorrow or worry). These manifestations (symptoms) are: lack of motivation for sexual activity, either by decreased or absent spontaneous desire (no sexual thoughts or fantasies) or by decreased or absent responsive desire to erotic cues and stimulation, inability to maintain desire or interest through sexual activity, loss of desire to initiate or participate in a sexual activity, including behavioral responses, such as avoidance of situations that could lead to sexual activity, which is not secondary to sexual pain disorders (3).

Specialized literature warns that testosterone has been frequently prescribed to women with low sexual desire, despite the lack of evidence of association between low testosterone levels and low libido and improvement in sexual dysfunctions when testosterone therapy is administered.

While serum androgen levels decline steeply in the early reproductive years, they do not fluctuate as a direct consequence of natural menopause (4).

Testosterone therapy is not routinely recommended for women with low androgen levels caused by adrenal insufficiency, hypopituitarism or surgical menopause, nor to enhance cardiometabolic parameters, cognitive health or to improve general wellbeing in healthy women. Therefore, the use of androgens in postmenopausal women remains controversial (5-9). In a systematic review, nine of ten studies failed to find a correlation between total testosterone levels and sexual desire, and four small studies showed little or none improvement in libido when compared to placebo (10). Therefore, the current position statement of the *Brazilian Society of Endocrinology and Metabolism* (SBEM) was developed to review the existing literature on the off-label use of testosterone to treat low sexual desire in women. Nine members of the Female Endocrinology and Andrology Department from SBEM were asked about it. The main results of this review can be summarized as follows (11):

- The relative hyperandrogenic status of menopause is associated with lower sex hormone binding globulin (SHBG) levels, resulting in increased free testosterone levels;
- Surgical menopause induced by bilateral oophorectomy, however, is associated with a different pattern: significantly lower total and free testosterone levels and dehydroepiandrosterone sulfate (DHEAS) decreased as compared with age-matched controls with both ovaries;
- In the presence of a proper HSDD diagnosis, once informed consent is obtained from the patient, an individualized trial of testosterone therapy for 3 to 6 months could be suggested, aiming midnormal premenopausal testosterone levels during treatment;
- In the absence of clear improvement and if adverse events are observed at 6 months, the use of testosterone should be ceased. There are no safety and efficacy evidences for testosterone therapy in women with HSDD beyond 24 months;
- Ideally, testosterone measurements should be obtained in the morning hours and in the follicular phase of the menstrual cycle. In normally cycling premenopausal women, testosterone levels should be measured at baseline and 3-6 weeks after starting treatment, especially to avoid supraphysiological concentrations, since response to therapy does not correlate with testosterone levels;
- Considering side effects and long-term safety, the role of testosterone in breast cancer and cardiovascular disease pathophysiology requires further elucidation;
- The abuse of androgens in sports and in the community, for aesthetic purposes, remains a major concern. Female athletes reported increased aggressiveness. Dyslipidemia, hypertension, arrhythmia, coagulation disorders, fibrosis, and cardiac hypertrophy have also been observed;
- There are currently no testosterone formulations approved for women by regulatory agencies in the United States, Brazil and most countries. Testosterone formulations approved for men are not recommended for women;
- When considering testosterone therapy, all risks and benefits should be thoroughly discussed with the patient before prescription.

This position statement was very well developed and leaves no doubt that female sexual dysfunction is a common complaint, specially in postmenopausal women, and may have a negative impact on quality of life. Testosterone seems to exert a positive effect on sexual desire in women with HSDD, however, requires caution and criteria in its management.

In addition to the above explained, there is a warning about cases of sexual problems in which testosterone therapy definitely does not work; for instance, when libido is impaired by the partner’s lack of attraction, resentment, anger, fear, embarrassment, or even by myths, taboos, misconceptions about sexual activity. In these cases, non-pharmacological methods are the therapeutic alternatives.
